# Psychoemotional Features of a Doubtful Disorder: Functional Dyspepsia

**Published:** 2012-09-25

**Authors:** D Dragos, O Ionescu, R Micut, DG Ojog, MD Tanasescu

**Affiliations:** * "Carol Davila" University of Medicine and Pharmacy, Bucharest, 1st Internal Medicine Department, University Emergency Hospital Bucharest, Romania; ** "Carol Davila" University of Medicine and Pharmacy, Bucharest

**Keywords:** personality inventory, psychological profile, psychological predisposition to disease

## Abstract

**Objective.** To delineate the psychological profile of individuals prone to FD-like symptoms (FDLS). **Method.** A triple questionnaire of 614 items (including psychological and medical ones) was given to 10192 respondents, the results were analyzed by means of Cronbach alpha, and Chi square test, together with an ad-hoc designed method that implied ranking and outliers detecting. **Results and conclusions.** FDLS appears to be an accompanying feature of many (if not most) human emotions and are more frequent in anxious, timid, pessimistic, discontent, irascible, tense, success-doubting, unexpected-dreading individuals, bothered by persistent thoughts and tormented by the professional requirements and the lack of time. A higher degree of specificity might have: chiefly fear of failure, susceptibility, and tension, secondarily emotivity, fear of unpredictable events, sense of insufficient time, preoccupation with authority factors, and tendency to endure unacceptable situations, and also faulty patience and lack of punctuality. Rumination appears to be the psychological tendency most strongly associated with FD. Nocturnal epigastric pain seems to indicate a submissive nature but a rather responsibilities-free childhood, while early satiety is associated with inclination to work and responsibility and preoccupation with self-image. The superposition of FD symptoms with biliary and esophageal symptoms cast a doubt over the distinctness and even the materiality of the various functional digestive disorders.

Abbreviations: ChiSq = chi-square; CrA = Cronbach alpha; OdRa = odds ratio; OdRaCL = OdRa confidence limits; E = exponential (for the sake of legibility we have used the exponential notation throughout this article; i.e. 4E-28 = 4×10-28); ErrProb = probability of error; SS = statistically significant; SD = standard deviation; a / m = the calculations were done by taking into account the average/ maximal score; P / M = psychological / medical category; PaMm / PmMa / PmMm / PaMa = the calculations were done by taking into account the average score for the PsyCt and the maximal score for the MedCt / the maximal score for PsyCt and the average score for the MedCt / and the maximal score for both / and the average score for both; R = the calculations were done for the FD_res category. FD = functional dyspepsia; FD_res / FD_ext = restricted / extended variant of the group of FD items; FDCt = FD category; FDLS = FD-like symptoms; MedCt / MedIt = medical category / item; PsyCt / PsyIt = psychological category / item;

## Introduction

There are many psychological associations attributed to FD and they are targeted by many relatively recent studies [**1-9**]. 

In previous articles [**10,11**] we have presented the rationale for conducting a cross-sectional study aimed at unraveling the psychoemotional correlations of the various internal disorders. We have also described the manner in which we have built our triple questionnaire (along with some psychological facts revealed by our study – many more are to follow).

## Material and Methods

Among the 614 items of our triple questionnaire (http://drdorindragos.ro/causesdiseases.html), 533 are exploring psychological issues [these are the psychological items (PsyIts)], while the remainder refers to various symptoms and diseases (respiratory, cardiac, digestive, urinary, gynecological) [we called these medical items (MedIts)]. The respondents were asked to fill in the items by checking one out of five variants (qualificatives) arranged in a quantitatively increasing order.

The PsyIts are divided into four main domains (the psychological domains), while the MedIts constitute into a fifth one (the medical domain). Each psychological domain includes several psychological categories (PsyCts), which are actually various psychological states/ tendencies (such as “Fear”, “Irritability”, “Desire to be appreciated” etc.). The medical domain includes several medical categories (MedCts), which are some of the most common disorders or fields of pathology (such as FD, renal diseases, ischemic heart disease, etc.). We have described in a previous paper [**1**] the way we have created the PsyIts. The MedIts were established based on the current disease definitions. Particularly, the FD-items were derived from the Rome III criteria [**12**].

The data analysis went through several stages

1. In order to establish the right assignment of items to categories we have systematically applied Cronbach alpha (CrA). We have done this for all the pairs of PsyIts (the results, primarily of psychological interest, will be presented in future papers). We have also applied it to groups of MedIts referring to the same pathological condition or to some related ones (i.e. for FD, some of the related medical conditions are esophageal and functional biliary disorders).

2. In order to establish the correlation between PsyIts/ PsyCts and MedIts/ MedCts we have used the chi square (ChiSq) test applied to all the pairs of items/categories including a psychological and a medical one. Therefore, this stage included 4 (2×2) substages: (2.1) MedCts with PsyCts, (2.2) MedCts with PsyIts, (2.3) MedIts with PsyCts, and (2.4) MedIts with PsyIts. While applying the ChiSq test, for the sake of clearness, we have reduced the five variants the respondent could check for each item to only two: below and above the average for the respective item (for each item we have calculated the average over the whole group of respondents after assigning numerical values – 1 through 5 – to the five qualificatives). For each two different items a two-by-two table emerged, to which the ChiSq test with one degree of freedom was applied. Due to the strength of our sample, there were no expected values less than 5, so we did not have to apply Fisher’s exact test. The correlation with an entire category was calculated in two different ways: taking into account either the highest (maximal) score of any item in that category (designated by “m” further in this paper), or the average (“a”) score of all the items in the category.

3. As the FD items seem to occupy the highest ranks among the correlations of most of the PsyIts, we assumed that this might be an unspecific effect – i.e. most emotional states induce FD-like symptoms (FDLS). In order to discover whether there are nevertheless PsyIts specifically associated with FD, we have looked for the PsyIts/ PsyCts that did not have FD among their strongest correlations. With the intention of giving some mathematical consistency to this search, we have ranked the various MedIts/ MedCts according to the strength of their association with each PsyIt/ PsyCt [evaluated in two ways: according to either the ChiSq value or the odds ratio (OdRa) value]. Thereafter, for any MedIt/ MedCt we have calculated the average and the standard deviation (SD) of its ranks over all the PsyIts/ PsyCts. We have then searched for the ranks that were further than 2 SDs from the average. We admit that this approach is partially flawed as the rank distribution is not normal (at least not for FD – it may be for other MedCts; for FD is very probably negatively skewed), but the worst that can happen are type II errors – this means that we might miss some truths, but at least we do not make false statements. Nonetheless, as most of the ranks for FD are very high, we are supposed to find some specific association not among the high ranks, but among the low ones – however, these correspond to OdRa values close to unity or below unity. Yet, only the OdRa values substantially below unity are clinically consequential and, as we shall see later, these values were duly highlighted by our method. The OdRa values close to unity mean weak association, if at all, between FD and the respective PsyIt/ PsyCt, so they are clinically irrelevant (whether we have overlooked them or not). To allow nonetheless for the possibility of committing a type II error, we have mentioned some of these results, which distinguish themselves by an OdRa value <1.2 and an ErrProb <10E-05 = 10-5 = 0.00001. Of course we have dismissed all the results with OdRaCL including the unity and with an ErrProb >0.05.

Regarding the use of CrA test, we remind that the generally accepted threshold for a significant CrA is 0.7. However, we have sometimes been more permissive by taking into consideration associations with a CrA as low as 0.6, if the correlated items were clearly semantically akin and they had no stronger associations with other items.

We have used several criteria to decide whether a group of items refers to the same thing (particularly to the same psychological/ medical condition):

1. The CrA for their group should preferably be at least 0.7 (the strong variant of the 1st criterion); a CrA below 0.7 (but not less than 0.6) was accepted if all the other criteria were fulfilled (the weak variant of the 1st criterion).

2. No item should significantly lower the CrA for the rest of the group; if an item does lower the CrA, this should preferably be with less than 0.02 (the strong variant of the 2nd criterion); a CrA lowering effect of at most 0.04 was accepted if all the other criteria were fulfilled (the weak variant of the 2nd criterion). To test for this criterion we have calculated the CrA for the group without the respective item.

3. The best association for each item should be with another item in the same group.

4. (only for the MedIts) The items should be medically related, i.e. they should refer to the same type of pathology.

Only the 4th criterion was considered mandatory, while the first three were applied somehow more flexibly. 

Regarding the use of ChiSq test, we reminded that we have explained it in a previous paper [1] the reason we have considered only the results with an error probability (ErrProb) <10E-07 (=10-7 = 0.0000001) [we called these results statistically significant (SS)]. Nonetheless, as mentioned above, for fear of sliding into type II error, we have occasionally granted some attention to results with an ErrProb >10E-07 but <10E-05 (= 10-5 = 0.00001) (relatively SS) and even to those with an ErrProb >10E-05 but <0.05 (marginally SS).

Our analysis included the answers from our first 10192 respondents (1711 M, 8481 F), aged 33.98 ± 11.39 yrs (35.43 ± 12.52 yrs for males, 33.68 ± 11.13 yrs for females).

## Results

What follows is a list of the terms used to designate some of the psychological categories: "Affectionate" = "Affectionate nature"; "Analyzing" = "Tendency to analyze before deciding"; "Author. parents" = "Authoritative parents"; "Authority" = "Preoccupation with authority factors"; "Cleanliness" = "Preoccupation with cleanliness"; "Commitment" = "Commitment, dedication"; "Communic. probl." = "Communication problems"; "Conflict" = "Tendency to conflict"; "Contemplative nat." = "Contemplative nature"; "Couple/partner" = "Couple, partner" = "Preoccupation with couple and/or partner"; "Demand. profess." = "Sense of demanding profession"; "Des. appreciation" = "Desire to be appreciated"; "Des. novelty" = "Desire for novelty"; "Dominating tend." = "Tendency to dominate, to impose oneself"; "Endure unaccept." = "Tendency to endure unacceptable things"; "Envied" = "Sense of being envied"; "Equity" = "Desire for equity, fairness, rectitude"; "Fear failure" = "Fear of failure"; "Fear unpredict." = "Fear unpredictable" = "Fear of the unpredictable"; "Fear" = "Fear of external threats"; "Feelings hiding" = "Tendency to hide one's feelings"; "Health" = "Preoccupation with health"; "House" = "Preoccupation with house, lodging, dwelling"; "Insuff. time" = "Sense of insufficient time"; "Irritability" = "Irritability, reactivity"; "Isolation" = "Tendency to isolation, withdrawal"; "Money" = "Preoccupation with financial security"; "Opression" = "Tendency to accept constraint, opression"; "Order" = "Preoccupation with order"; "Perfectionism" = "Thoroughness, perfectionism"; "Profession preocc." = "Preoccupation with profession"; "Prudence" = "Prudence, precaution"; "Quarrel. parents" = "Quarreling parents"; "Des. relaxation" = "Desire for/ tendency to relaxation"; "Rumination" = "Rumination, tendency to persistent thoughts"; "Self-assertion" = "Preoccupation with self-assertion"; "Self-doubt" = "Self-doubt, lack of self-confidence"; "Self-image" = "Preoccupation with self-image"; "Self-value" = "Preoccupation with proving one's value"; "Tension" = "State of tension, strain"; "Time efficiency" = "Desire for time efficiency"; "Work" = "Propensity to work, activity"; 

Stage 1 – the right assignment of the medical items

Among the FD items, the only one that does not meet our 2nd criterion is ”I feel quickly full while eating, so that I cannot finish a normal-sized meal” (it is marked with a “§” in the following table) – this means that CrA is lower for the group of FD items with this item than without it (**Table 1**), suggesting that this item might actually not refer to the same type of pathology as the other items in the group. CrA for groups of items is displayed in the next two tables. CrA for the whole group is indicated in the row immediately below the title row (it corresponds to “None” in the column headed “The missing item”), while CrA for the group without one of the items is indicated in the same row with the respective item.

**Table 1:** CrA for the extended FD group and for the group without one of the items (“§” marks the item that lowers the group CrA)

The missing item	CrA
None	0.77
”I feel pain (aching, burning etc.) in the mid upper abdomen, not relieved by passing stool or gas”	0.74
”I have an unpleasant feeling of fullness/ discomfort after eating”	0.75
”When I am hungry, I have a stomach ache (upper abdominal, in the middle), which disappears after eating”	0.73
”When I am hungry, my stomach hurts (in the mid upper abdomen)”	0.72
”When I am nervous my stomach hurts (in the mid upper abdomen)”	0.75
”I have a stomach ache (pain in the mid upper abdomen) after eating”	0.73
”I wake up during night with stomach aches (mid upper abdominal pains)”	0.76
§ ”I feel quickly full while eating, so that I cannot finish a normal-sized meal”	0.8

After eliminating the above-mentioned item, the remaining ones still fulfill the 2nd criterion (**Table 2**), therefore we may conjecture that they constitute a homogeneous group reflecting the same pathological condition.

**Table 2:** CrA for the restricted FD group and for the group without one of the items

The missing item	CrA
None	0.8
”I feel pain (aching, burning etc.) in the mid upper abdomen, not relieved by passing stool or gas”	0.77
”I have an unpleasant feeling of fullness/ discomfort after eating”	0.79
”When I am hungry, I have a stomach ache (upper abdominal, in the middle), which disappears after eating”	0.77
”When I am hungry, my stomach hurts (in the mid upper abdomen)”	0.74
”When I am nervous my stomach hurts (in the mid upper abdomen)”	0.79
”I have a stomach ache (pain in the mid upper abdomen) after eating”	0.76
”I wake up during night with stomach aches (mid upper abdominal pains)”	0.79

The 1st criterion is obviously fulfilled by both groups. We have implicitly satisfied the 4th criterion by choosing our items according to the Rome III criteria for FD. Nevertheless, there were some problems with the last remaining criterion, the 3rd: some items fulfilled it (i.e. their best correlation was with another FD item) (**Table 3**), others did not, their best correlations being with items pertaining to other MedCts (to esophageal or functional biliary disorders) (**Table 5**). However, we kept these items in the FD category (FDCt), as they met the other three criteria.

**Table 3:** FD items whose best correlations are with items in the same MedCt (i.e. with another FD item)

The correlated FD items:	CrA
(FD) ”When I am hungry, I have a stomach ache (upper abdominal, in the middle), which disappears after eating”	(FD) ”When I am hungry, my stomach hurts (in the mid upper abdomen)”	0.84
(FD) ”I have an unpleasant feeling of fullness/ discomfort after eating”	(FD) ”I have a stomach ache (pain in the mid upper abdomen) after eating”	0.63
(FD) ”When I am nervous my stomach hurts (in the mid upper abdomen)”	(FD) ”When I am hungry, my stomach hurts (in the mid upper abdomen)”	0.6
(FD) ”When I am hungry, I have a stomach ache (upper abdominal, in the middle), which disappears after eating”	(FD) ”I have a stomach ache (pain in the mid upper abdomen) after eating”	0.53

**Table 4:** FD items whose best correlations are with items in other MedCts: esophageal (esoph.) or functional biliary (funct. bil.) disorders. For each item, the FD item they are best correlated with is also mentioned

The item to be correlated with	CrA
The correlation of ”I feel pain (aching, burning etc.) in the mid upper abdomen, not relieved by passing stool or gas” with:
(esoph.) I have pains in the epigastrium (just below the breastbone) minutes or hours after eating	0.76
(funct. bil.) I have mid- or right-upper abdominal pain lasting for at least 30 minutes, building up to a steady level and not relieved by passing stool or gas, by position change or by ulcer medication (Maalox, Omeprazole, Ranitidine etc.)	0.72
(esoph.) If I eat too much, after a few minutes or hours I have heartburn (a burning sensation in the chest rising up behind the breastbone)	0.69
(FD) ”I have a stomach ache (pain in the mid upper abdomen) after eating”	0.68
The correlation of ”I have a stomach ache (pain in the mid upper abdomen) after eating” with:
(esoph.) I have pains in the epigastrium (just below the breastbone) minutes or hours after eating	0.76
(esoph.) A few minutes or hours after eating too much, I feel pain just below the breastbone	0.72
(esoph.) If I eat too much, after a few minutes or hours I have heartburn (a burning sensation in the chest rising up behind the breastbone)	0.69
(esoph.) I have heartburn (a burning sensation in the chest rising up behind the breastbone) within a few minutes or hours after a meal	0.69
(FD) ”I feel pain (aching, burning etc.) in the mid upper abdomen, not relieved by passing stool or gas”	0.68
The correlation of ”I wake up during night with stomach aches (mid upper abdominal pains)” with:
(esoph.) I wake up at night with heartburn (a burning sensation in the chest rising up behind the breastbone)	0.73
(esoph.) If I eat too much in the evening, I wake up at night with heartburn (a burning sensation in the chest rising up behind the breastbone)	0.63
(funct. bil.) I have mid- or right-upper abdominal pain lasting for at least 30 minutes, building up to a steady level and not relieved by passing stool or gas, by position change or by ulcer medication (Maalox, Omeprazole, Ranitidine etc.)	0.61
(FD) ”I feel pain (aching, burning etc.) in the mid upper abdomen, not relieved by passing stool or gas”	0.61
The correlation of ”I feel quickly full while eating, so that I cannot finish a normal-sized meal” with:
(funct. bil.) After fat or fried food meals, I have nausea and/ or vomiting	0.27
(FD) ”When I am hungry, my stomach hurts (in the mid upper abdomen)” 0.25	0.25

There is still another problem with our troublesome item (“I feel quickly full while eating, so that I cannot finish a normal-sized meal”), as its correlations with any other item are very weak. That is why, in our further analysis, we have made separate calculations for the group of FD items with and without this item – we called these groups FD_ext and FD_res, respectively (“ext” from extended, “res” from “restricted”). The analysis revealed some sizable differences between these two groups.

**Stage 2 – establishing the correlation between psychological and the medical items/categories**

Among the item-to-item correlations there are thousands with an ErrProb <10E-07 (i.e. 10-7), therefore it is impossible to present all of them. What struck us was that some of the best correlations of almost any PsyIt were with MedIts in the FDCt. Similarly, among the correlations of the various PsyIts with MedCts, the MedCt of FD was usually in a leading position. The same was true about the correlations of the various PsyCts with MedCts – in most cases the correlation with FDCt was among the strongest.

The next table (**Table 5**) displays the correlations of FD items taken as a group with the various PsyCts, the calculations being made by taking into account the average score for both the PsyCt and the FDCt. We have made calculations separately for FD_res and FD_ext. For a more clear view, we have merged the two corresponding tables side by side.

**Table 5:**
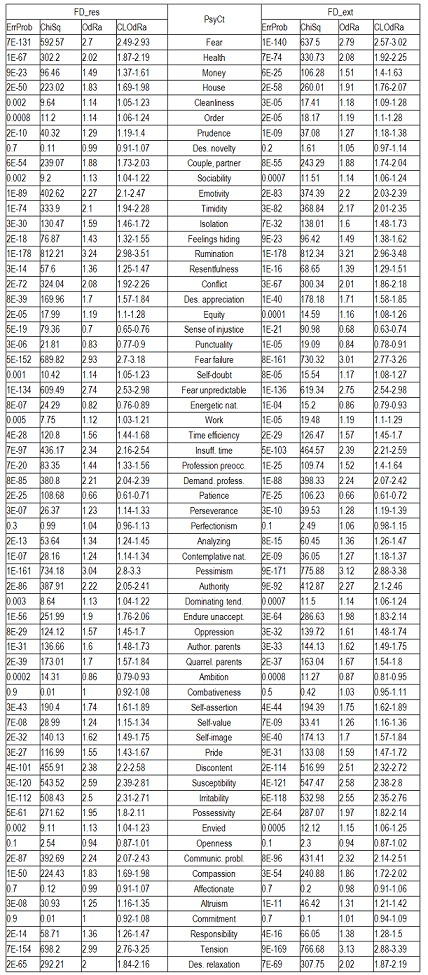
Correlation of FD with the various PsyCts, taking into account the average score for both (PaMa). Separate calculations were made for the restricted (FD_res) and the extended (FD_ext) variants of the FD group of items.

**Fig 1:**
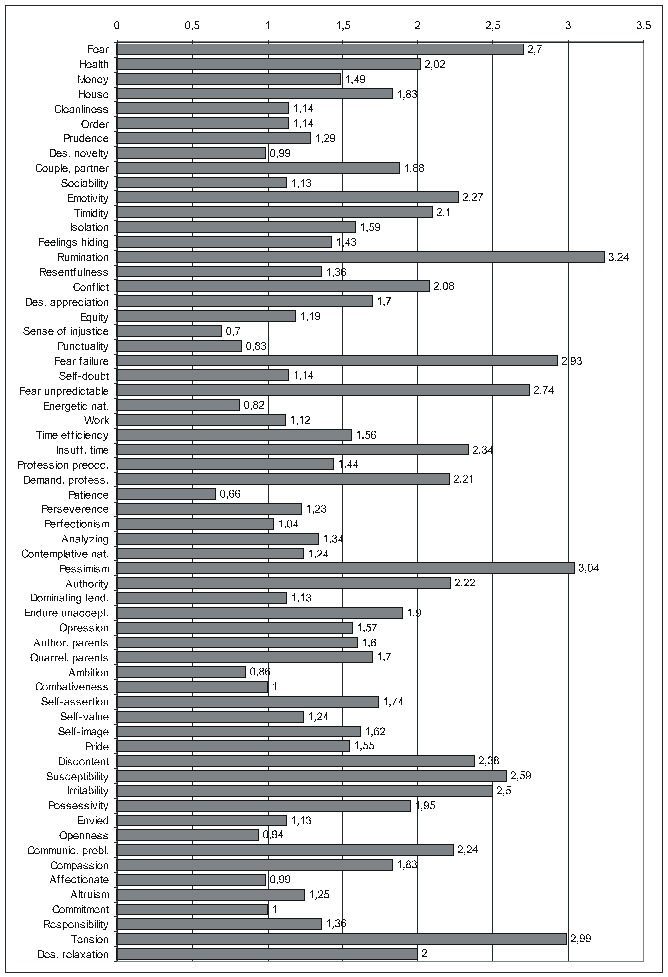
Correlation of FD_res with the various PsyCts, taking into account the average score for both (the numbers labeling each bar represent the odds ratio values).

**Fig 2:**
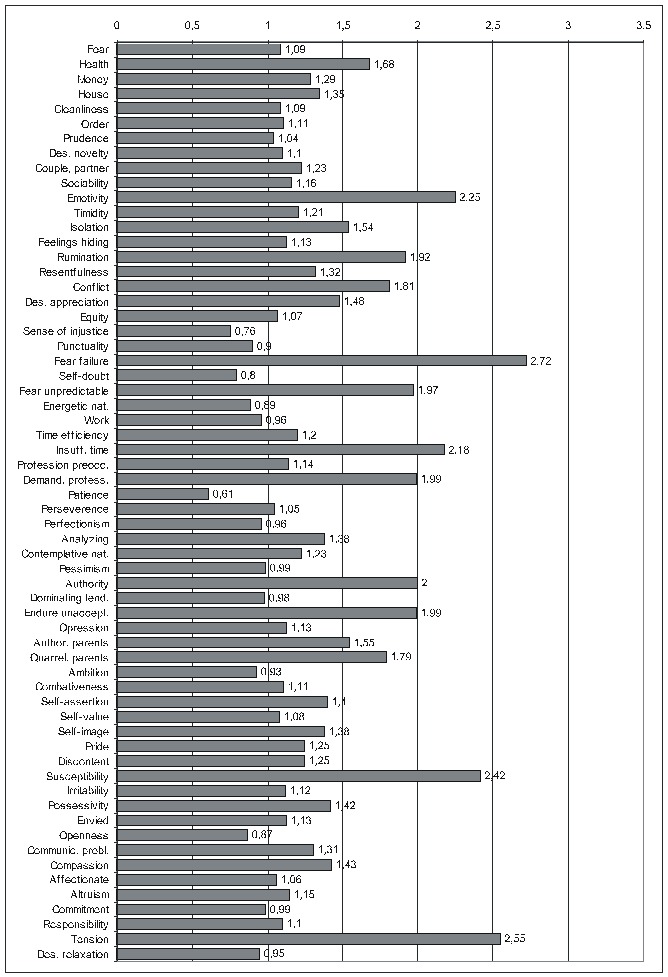
Correlation of FD_res with the various PsyCts, taking into account the maximal score for the PsyCt and the average score for FD_res (the numbers labeling each bar are the odds ratio values).

The purpose of the first of the two ensuing diagrams (**Fig. 1**) is to facilitate the understanding of the previous table – it only refers to FD_res. The second one ((**Fig. 2**) illustrates the results obtained by using the maximal score for the PsyCt and keeping the average score for FD_res.

Regarding the other ways of making the calculations, as there is not enough space to present all the results extensively, we shall display only the most important information (**Table 6**): the ErrProb and the OdRa, leaving out the ChiSq value and the confidence limits for OdRa (OdRaCL) (generally, the results with an ErrProb < 0.05 correspond to OdRaCL including the unity – meaning lack of statistical significance).

**Table 6:**
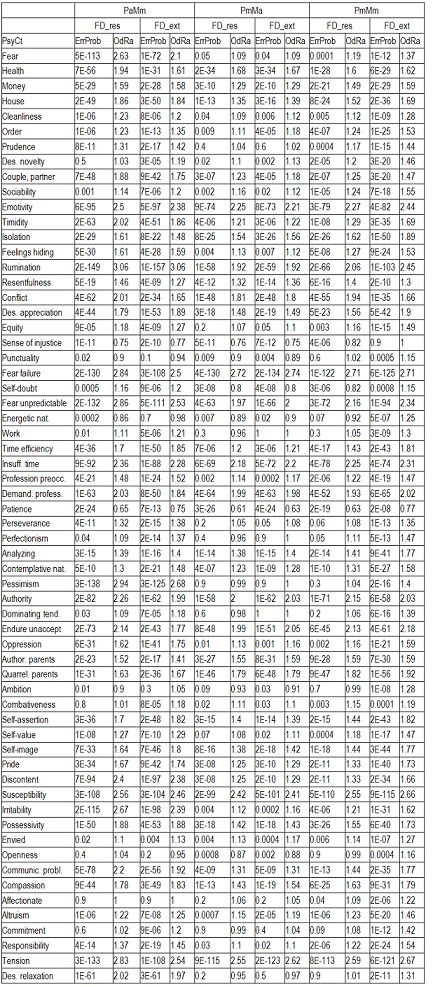
Correlation of FD (both FD_res and FD_ext) with the various PsyCts, taking into account the average score for PsyCts and the maximal score for the MedCt (PaMm), the maximal score for PsyCts and the average score for the MedCt (PmMa), and the maximal score for both (PmMm).

**Fig. 3:**
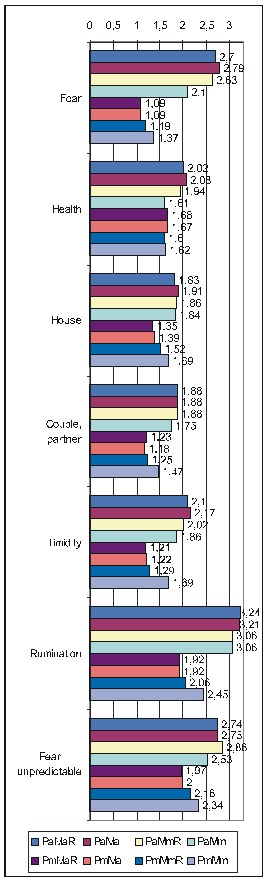
Comparative OdRa demonstrating the higher values obtained by averaging the scores for the PsyCts (top four bars in each group). For many PsyCts, among the bottom four bars, the bottom most one is also strikingly longer than the other three [**1**].

**Fig. 4:**
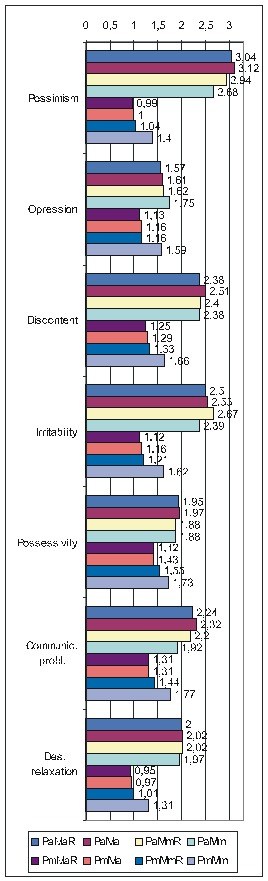
Comparative OdRa demonstrating the higher values obtained by averaging the scores for the PsyCts (top four bars in each group). For many PsyCts, among the bottom four bars, the bottom most one is also strikingly longer than the other three [**2**].

**Fig. 5:**
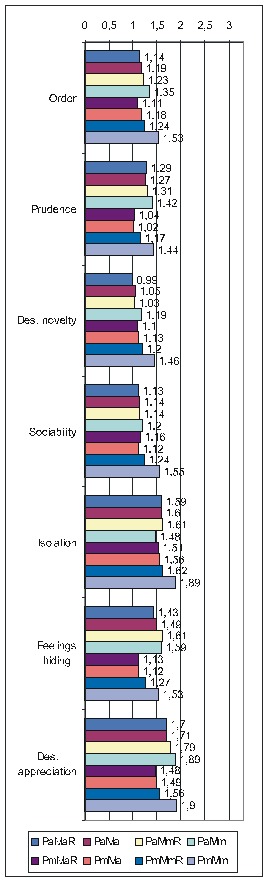
Comparative OdRa demonstrating that, among the values obtained by using the maximal score for the PsyCts (bottom four bars in each group), the value obtained by also taking into account the maximal score for FD_ext (the bottom most bar in each group) is evidently higher [**1**].

**Fig. 6:**
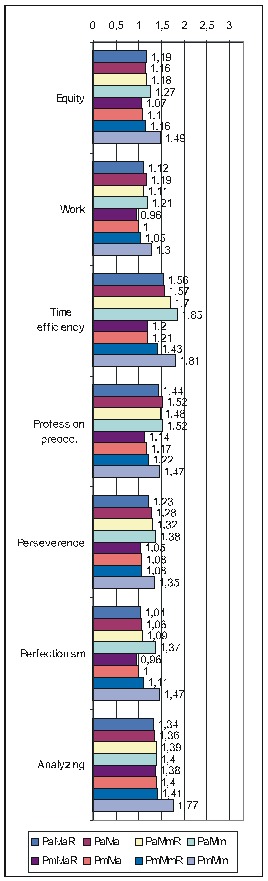
Comparative OdRa demonstrating that, among the values obtained by using the maximal score for the PsyCts (bottom four bars in each group), the value obtained by also taking into account the maximal score for FD_ext (the bottom most bar in each group) is evidently higher [**2**].

**Fig. 7:**
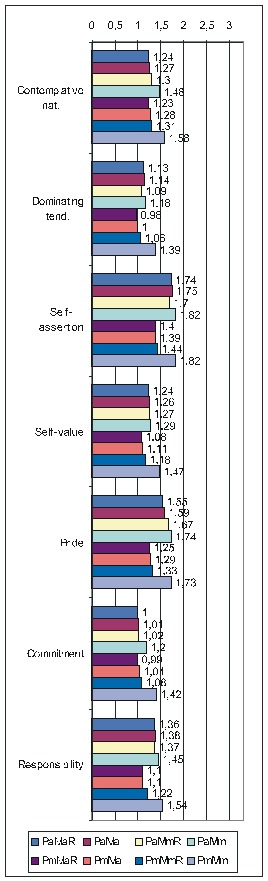
Comparative OdRa demonstrating that, among the values obtained by using the maximal score for the PsyCts (bottom four bars in each group), the value obtained by also taking into account the maximal score for FD_ext (the bottom most bar in each group) is evidently higher [**3**].

**Fig. 8:**
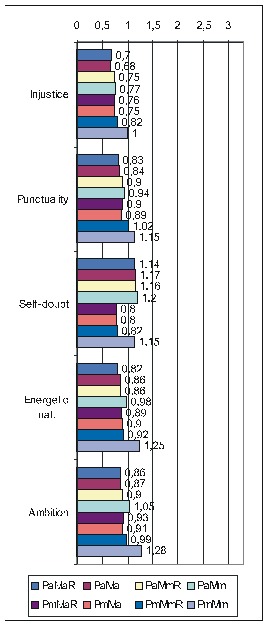
Comparative OdRa demonstrating that, among the values obtained by using the maximal score for the PsyCts (bottom four bars in each group), the value obtained by also taking into account the maximal score for FD_ext (the bottom most bar in each group), is not only higher, but it also qualitatively changes (even reverses) the correlation between the PsyCt and FD (the OdRa from subunitary becomes unitary or even supraunitary).

As we hardly expect anyone to attentively and eagerly study the previous table, we attempt to ease its understanding by the following diagrams. For lack of space, we have chosen only the most representative correlations. For each PsyCt, the top four bars reflect the OdRa values obtained by averaging the scores for the PsyCt (“Pa”) – for many of the PsyCts, these bars are strikingly longer than the other four. The first two diagrams (**Fig. 3** and **Fig. 4**) illustrate this feature. Another conspicuous characteristic is that for many PsyCts, among the bottom four bars (obtained by taking into account the maximal score for the PsyCt – “Pm”), the bottom most one (the one corresponding to calculating the OdRa by taking into account the maximal score for both the PsyCt and FD_ext – “PmMm”) is strikingly longer than for the other three bars. The next three diagrams (**Fig. 5**, **Fig. 6**, and **Fig. 7**) illustrate this second feature. For several PsyCts, this feature is exaggerated to the point that the OdRa value corresponding to the bottom most (“PmMm”) bar is not only longer, but also qualitatively changes (even reverses) the correlation between the PsyCt and FD (the OdRa from subunitary becomes unitary or even supraunitary) – the last diagram (**Fig. 8**) illustrates this last feature.

**Stage 3 – searching for ranking outsiders**

Most of these results are not SS, therefore they are not worth mentioning. We shall present only the SS ones. That is why no results produced by investigating the item-to-category or by category-to-item correlations are displayed (no one was SS). SS results have been derived only from category-to-category (i.e. PsyCt to FDCt) and from item-to-item (i.e. PsyIt to FD item) correlations.

Category-to-category (i.e. PsyCt to FDCt) correlations 

This yielded a single marginally significant correlation, a little bit improved when the calculations were done for FD_ext by averaging the score for both the PsyCt and the FDCt (**Table 7** and **Table 8**).

**Table 7:**
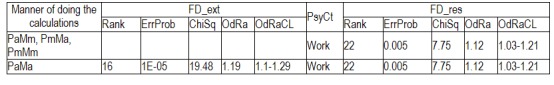
Searching for ChiSq-ranking outsiders among the PsyCt-to-FDCt correlations, (missing values mean not SS values)

**Table 8:**
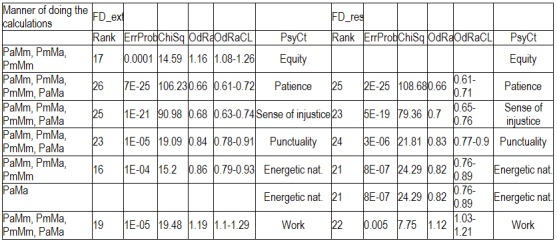
Searching for OdRa-ranking outsiders among the PsyCt-to-FDCt correlations, (missing values mean not SS values)

Item-to-item (i.e. PsyIt to FD item) correlations

The Item-to-item correlations were more productive (**Table 9**). 

**Table 9:**
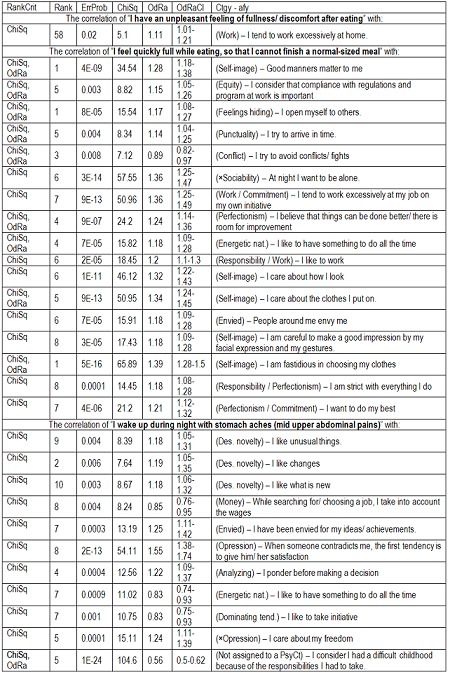
Searching for ChiSq- and OdRa-ranking outsiders among the correlations of PsyIts with FD items (RankCrit = ranking criterion, “×” means that the opposite of that item was taken into account)

## Discussions

Many PsyCts are associated with FD – actually too many, so that it is difficult to perceive a central feature. Moreover, FD seems to have a leading position among all the other disorders in its correlations with the various PsyCts. Therefore, should FDLS be considered the manifestations of a distinct disease (i.e. FD) or are they a mere physical (or rather physiological) extension of the affects (more precisely, the most frequently encountered one)? Concordant with this, we should remind that there are voices speaking against the utility of the Rome criteria [**12**].

Some of the strongest associations are with the psychological disorders most intensely studied in the quest for the psychoemotional foundations of the diseases: fear (an equivalent of anxiety), pessimism (which stands for depression), and tension/ strain.

Interestingly, the strongest association is with ”rumination”, a result which, to our knowledge, has not been obtained previously. In view of this correlation, we would have expected a tighter association with “resentfulness”. The discrepancy arises probably from the moral burden of rancor, and therefore the general tendency to deny it. 

There are strong associations with ”fear of failure” and ”fear of the unpredictable” but, quite surprisingly, not with ”self-doubt”. One probable explanation is that people tend to deny their lack of self-confidence when questioned about it in general terms, but acknowledge it when asked about specific situations that are bound to reveal it, such as sustaining an examination, attending an interview, starting something new, bringing an enterprise to an end, etc.

Some of the common worries (for house, health, and couple/ partner) have also good correlations with FDLS.

Discontent, susceptibility, and irritability constitute into a group of related PsyCts strongly associated with FDLS. The latter (and probably the former) are well correlated also with the tendency to conflict.

There is also a good association with the timidity-emotivity group, and thereto probably related is the association with FDLS of some other PsyCts, such as preoccupation with authority factors, inclination to endure unacceptable situations, and communication problems. A relation with the unadmitted faltering self-confidence may also be suspected.

Some disparities are also interesting: the correlations with “Insuff. time” and “Demand. profess.” are far better than those with “Time efficiency” and “Profession preocc.” respectively. These individuals are not adamant to make the most of their time or to fulfill their professional duties, but they feel oppressed by the lack of time and by the exigencies of their job. It is a self-centered attitude, to be contrasted with an outward directed one, apt to distance the individual from his discontents, failings, and fears. We suggest that this propensity concentrates on discomfort-generating psychological states may be pathophysiologically related to the excessive attention directed toward the internal sensations.

Some of the significant negative correlations are worth mentioning, most of them being coherent with the above discussed ones. The lack of punctuality and patience reminds of the sense of insufficient time. The weak ambition is understandable in a self-doubting, test-shunning, unforeseen-apprehending individual. Moreover, this is hardly the person we expect to have an energetic nature. What is interesting is the negative association with the sense of injustice: FDLS-individuals tend not to view their mishaps as undeserved blows of an unfair fate, probably because their attention is primarily focused on avoiding putative sources of discomfort, which they regard not as links in the chain of an undeservedly punishing master-plan, but rather as random, unforeseeable occurrences, whose anticipation brings them to a timorous, fearful attitude.

Rather intriguing are the dissimilarities between the results obtained by using the maximal versus the average scores for the PsyCts – for some of them the average scores based calculations engendered far better correlations than those produced by using the maximal scores. The most striking differences were for fear, timidity, rumination, pessimism, discontent, irritability, communication problems, and relaxation tendency. Some of them are (almost) completely wiped out [namely, the OdRa drops (almost) to unity]: fear, pessimism, irritability, relaxation tendency. It is probably no coincidence that these are some of the most general psychological tendencies, therefore, some of the most frequent ones, but also some of the least expected to be specifically associated with a given medical condition. The leveling effect of the maximal score based calculations arises precisely from the high prevalence of these psychological states: most individuals are bound to have at least one of their various manifestations, and that will be reflected in a high score for the corresponding PsyIt, therefore in a high score for the entire PsyCt. Thus, we may conjecture that the psychological tendencies more specifically associated with FD are primarily fear of failure, susceptibility, and tension, but also emotivity, fear of unpredictable events, sense of insufficient time, preoccupation with authority factors, and the tendency to endure unacceptable situations.

The results obtained by ranking the PsyCts-to-FDCt correlations corroborate the above-mentioned ones, pointing out the negative correlation with patience, punctuality, energetic nature, and sense of injustice.

At their turn, the results obtained by ranking the correlations of PsyIts to FD items concur with the same conclusions, but add some new, potentially interesting, ones. There are actually only three PsyIts involved in these correlations. One of them yielded a single result, which is marginally SS – we would rather dismiss it. All the other correlations are generated by only two other PsyIts, one belonging to FD_res [let us call it the “insider”: ”I wake up during night with stomach aches (mid upper abdominal pains)”], the other being our outsider item (”I feel quickly full while eating, so that I cannot finish a normal-sized meal”) which was banished from the elitist FD_res into the more tolerant FD_ext.

Referring to the insider, only two of its correlations are absolutely SS, one suggesting a submissive tendency (“When someone contradicts me, the first tendency is to give him/ her satisfaction”), the other indicating, rather surprisingly, a negative association with a difficult childhood. While the first is congruent with the general psychological picture of the FD person outlined until now (timid, fearful etc.), the second may seem to contradict what was known about the association of FD with abuses in childhood. On the other hand, assuming responsibilities is quite different from being abused. Among the relatively SS correlations, there are some consistent with the submissive nature (negative correlation with “I care about my freedom” and “I like to take initiative”), the lack of energy (negative correlation with “I like to have something to do all the time”), and the fearful nature (the hesitancy suggested by “I ponder before making a decision”), others are not (there are three PsyIts indicating an appetite for change and novelty, which is rather unexpected in an anxious person).

FD persons (at least those with nocturnal epigastric pains) tend to have a compliant nature, but are not distressed by the memory of a responsibilities-ridden childhood.

Some of the item-to-item correlations involving the “outsider” are in contradiction with the features suggested by the correlations of the other items (expressively illustrated by the FD_ext bar popping out in many of the diagrams from the “Results” section). Is it the consequence of this item not actually belonging to the FD picture or, on the contrary, this is precisely the item that could make FD a definite disorder, and not only the upper abdominal chunk from the vast collection of unspecific symptoms elicited in the wake of emotional turmoils?

Referring to the absolutely SS correlations of the outsider item, the preoccupation with self-image was also pointed out by the category-to-category correlations, but the desire to work is contradictory to the psychological image built so far. Consonant with it are some less significant correlations expressing commitment and perfectionism, energetic nature, and inclination to work and responsibility.

The psychological features that were better (or even exclusively) highlighted by using the average scores in our calculations are also those having some of the highest OdRa values and these are the emotional disturbances most widely recognized as being associated with various diseases. This is probably another argument for the unspecific nature of FDLS. We may push even further our conclusions to the point of asking ourselves whether there is such a condition as FD or this is only a label put to the subset of bodily sensations that most frequently accompany psychological unease. The overlap of the FD, esophageal, and functional biliary items supports the same idea, as it rises a question mark about how distinct actually are these three types of functional digestive disorders.

In the case we still abide by the reality of this diagnosis, early satiety might be its best clinical marker, and to a lesser degree nocturnal epigastric pain. Alternatively, these two symptoms may describe two varieties of the same disorder (or perhaps two different disorders), one more frequent in reliable hard-working persons, the other in obedient ones. 

We should stress out that this is in line with some of the current trends in FD research – many gastroenterologists are inclined to view it as a cover for several distinct disorders (such as epigastric pain syndrome, postprandial distress syndrome, cyclic vomiting syndrome, chronic idiopathic nausea) that might differ from one another by their symptoms and mechanisms [**12**] – or possibly by their psychoemotional background, would be our suggestion.

## Conclusions

Many psychological tendencies are associated with FDLS, especially anxiety, timidity, pessimism, discontent, irritability, rumination, tension, professional strain, time pressure, fear of failure and of unanticipated occurrences. Among this, rumination seems to hold the leading position. 

More specific in their association with FD may be fear of failure, susceptibility, and tension, together with emotivity, fear of unpredictable events, sense of insufficient time, preoccupation with authority factors, and compliance with unbearable situations. FD individuals are lacking patience and punctuality; they do not have an energetic nature, and do not feel wronged by others.

Nocturnal epigastric pain is more frequent in obeisant individuals with a duty-free childhood, while early satiety occurs more often in dependable hard-working ones that care about the impression they make on others.

Many FD items are well correlated with those pertaining to biliary and esophageal disorders blurring the boundaries between the various functional digestive disorders and even their factuality. 
